# Application of Membrane Capacitive Deionization as Pretreatment Strategy for Enhancing Salinity Gradient Power Generation

**DOI:** 10.3390/membranes15020056

**Published:** 2025-02-08

**Authors:** Seoyeon Lee, Juyoung Lee, Jaehyun Ju, Hyeongrak Cho, Yongjun Choi, Sangho Lee

**Affiliations:** 1 School of Civil and Environmental Engineering, Kookimin University, 77 Jeongneung-ro, Seongbuk-gu, Seoul 02707, Republic of Korea; yeon623@kookmin.ac.kr (S.L.); marks2@kookmin.ac.kr (J.L.); rhino@kookmin.ac.kr (H.C.); choiyj1041@kookmin.ac.kr (Y.C.); 2 Korea Testing Laboratory, 10, Chungui-ro, Jinju-si 52852, Republic of Korea; jhju@ktl.re.kr; 3 Water Technologies Innovation Institute and Research Advancement (WTIIRA), Saudi Water Authority (SWA), Al-Jubail 31951, Saudi Arabia

**Keywords:** salinity gradient power, pressure-retarded osmosis, reverse electrodialysis, membrane capacitive deionization, brackish water reverse osmosis brine, power density, membrane fouling

## Abstract

Salinity gradient power (SGP) technologies, including pressure-retarded osmosis (PRO) and reverse electrodialysis (RED), have the potential to be utilized for the purpose of harvesting energy from the difference in salinity between two water streams. One challenge associated with SGP is a reduction in power density due to membrane fouling when impaired water is utilized as a low-salinity water stream. Accordingly, this study sought to explore the feasibility of membrane capacitive deionization (MCDI), a low-energy water treatment technique, as a novel pretreatment method for SGP. Laboratory-scale experiments were conducted to evaluate the impact of MCDI pretreatment on the performance of PRO and RED. The low-salinity water was obtained from a brackish water reverse osmosis (BWRO) plant, while the high-salinity water was a synthetic seawater desalination brine. The removal efficiency of organic and inorganic substances in brackish water reverse osmosis (BWRO) brine by MCDI was estimated, as well as theoretical energy consumption. The results demonstrated that MCDI attained removal efficiencies of up to 88.8% for organic substances and 78.8% for inorganic substances. This resulted in a notable enhancement in the lower density for both PRO and RED. The power density of PRO exhibited a notable enhancement, reaching 3.57 W/m^2^ in comparison to 1.14 W/m^2^ recorded for the BWRO brine. Conversely, the power density of RED increased from 1.47 W/m^2^ to 2.05 W/m^2^. Given that the energy consumption by MCDI is relatively low, it can be surmised that the MCDI pretreatment enhances the overall efficiency of both PRO and RED. However, to fully capitalize on the benefits of MCDI pretreatment, it is recommended that further process optimization be conducted.

## 1. Introduction

Increasing energy demand and environmental concerns have led to the need for a transition to sustainable and renewable energy technologies. Since the onset of the Industrial Revolution, the combustion of fossil fuels for energy has notably increased CO_2_ emissions, thereby disrupting the natural carbon cycle and accelerating global warming. In 2023, total energy-related CO_2_ emissions increased by 1.1%, reaching a record high of 37.2 Gt CO_2_ (International Energy Agency [IEA]) [1]. This persistent increase in CO_2_ emissions has resulted in a total temperature rise of approximately 1.2 °C since pre-industrial times. Crossing the one-degree threshold represents a pivotal milestone, as it places us more than halfway towards the 2 °C warming limit set to prevent the most severe impacts of climate change [2]. In response to this challenge, renewable energy sources have become a central focus of climate change mitigation strategies.

Among these, salinity gradient power (SGP), also known as blue energy, stands out as a promising and cost-effective marine renewable energy source [3]. SGP, which is generated by mixing high-salinity water with low-salinity water (e.g., seawater and river water) has been known for a long time since it was first introduced in 1953 [4]. It can reduce global greenhouse gasses emissions by 8 Gt CO_2_-eq/year by replacing coal-fired power plants—approximately 21% of current emissions from fossil fuels. This corresponds to reductions of 25%, 27%, and 8% in energy-related emissions of CO_2_, CH_4_, and N_2_O, respectively [5]. SGP represents viable approaches to reduce greenhouse gas emissions and diversify the renewable energy portfolio.

SGP systems primarily operate using two methods: pressure-retarded osmosis (PRO) and reverse electrodialysis (RED) [6,7,8,9]. While PRO relies on osmotic pressure to drive water through a semipermeable membrane [10], RED utilizes the ion movement through ion-exchange membranes to produce electric power [11]. In PRO, freshwater permeates through a semipermeable membrane into highly concentrated seawater [12]. This process increases the pressure on the seawater side, which can then be utilized to drive turbine blades and produce electricity [13]. In contrast, RED relies on the movement of ions across alternating cation and anion exchange membranes (CEMs and AEMs) arranged in a stack [14]. These membranes separate compartments filled alternately with seawater and freshwater [15]. The energy generated during the mixing of fresh and saline water is derived from the Gibbs free energy of mixing [16].

Despite their potential, the efficiency of SGP processes is often hindered by membrane fouling and scaling, particularly when using impaired water sources as feed solutions [17,18,19,20]. As with other membrane-based technologies, fouling remains an unavoidable challenge for both types of SGP process [21,22]. Fouling not only reduces power density but also escalates operational and maintenance costs, limiting the large-scale implementation of SGP technologies [23,24]. Conventional methods to mitigate fouling, such as chemical cleaning or advanced pretreatment techniques, can be resource-intensive and environmentally detrimental [25]. Furthermore, the energy consumption associated with fouling control measures should be minimized in order to render the SGP process economically viable [26]. This underscores the need for innovative, cost-effective, and energy-efficient strategies to address membrane fouling and scaling in SGP applications.

Membrane capacitive deionization (MCDI) is a novel electrochemical desalination technology [27,28] that has potential as a novel pretreatment strategy for fouling mitigation in SGP process [29]. By utilizing electrically charged electrodes to remove ions and organic substances from feed water, MCDI reduces fouling propensity in downstream processes [30,31]. The low energy consumption and operational simplicity of MCDI make it an attractive option for integration into hybrid systems, enhancing the overall efficiency and sustainability of SGP technologies [32]. Recent studies have demonstrated the potential of MCDI to reduce fouling and scaling [33], yet its application in salinity gradient power systems remains underexplored.

This study investigates the integration of MCDI as a pretreatment step for PRO and RED systems using brackish water reverse osmosis (BWRO) brine as the feed solution. Laboratory-scale experiments were conducted to evaluate the performance improvements achieved through MCDI pretreatment. Key performance indicators, including power density, ion removal efficiency, and specific energy consumption, were analyzed to quantify the benefits of this hybrid approach. Additionally, the impact of varying operational parameters, such as flow rate and applied voltage, was assessed to optimize the MCDI process for SGP applications. The findings of this study highlight the feasibility and effectiveness of MCDI as a pretreatment strategy to enhance the energy efficiency and operational stability of SGP systems.

## 2. Materials and Methods

### 2.1. Feed Solutions

A low TDS (total dissolved solid) and a high TDS solution were used as the feed solutions to the PRO and RED processes. The sources and information for these solutions are described below.

#### 2.1.1. Low TDS Solution (BWRO Brine)

The BWRO brine utilized in this study was provided by a domestic wastewater reuse plant in the Republic of Korea. The reuse plant receives wastewater previously treated at a municipal wastewater treatment plant and reprocesses it for subsequent use in industrial applications. With a total treatment capacity of 90,000 m^3^/day, the reuse plant primarily uses BWRO membranes to remove ions and dissolved organic matters. The concentrate capacity of the facility is 31,210 m^3^/day, which is returned back to the wastewater treatment plant. In this study, total organic carbon (TOC), ultraviolet-visible (UV-254), and suspended solids (SSs) were measured in the laboratory, while anions and cations were analyzed by another agency using inductively coupled plasma mass spectrometry (ICP-MS) and inductively coupled plasma (IC). The overall water quality is summarized in Table 1.

#### 2.1.2. High TDS Solution (Synthetic SWRO Brine)

The high TDS solution was prepared by dissolving sodium chloride (Sigma-Aldrich, St. Louis, MO, USA) in deionized water at a concentration of 1.2 M, resulting in an electrical conductivity of 101 mS/cm. It was postulated that this solution simulates the brine from a seawater desalination plant, referred to as SWRO brine. The high TDS solution was used as a draw solution in the PRO process and as a concentrated saline stream in the RED process. The fouling potential on the high TDS solution (draw) side was not considered because it was assumed to be less important than that on the low TDS solution (feed) side.

### 2.2. Lab-Scale MCDI System

#### 2.2.1. MCDI Module

The specifications of the electrodes, ion exchange membranes, and spacers are summarized in Table 2. The electrode, ion exchange membranes, and spacer were purchased from Pureechem Co. (Cheongju-si, Republic of Korea). The dimensions of the activated carbon electrode are 100 mm in width and 100 mm in length, with an adsorption capacity of more than 16 mg/g. The ion exchange membranes, made of polyethylene, have the same dimensions as the electrode. The spacer, composed of polyethylene terephthalate, is 110 mm wide and 110 mm long. A hole was drilled in the upper left corner of the electrode, in the protruding part (2.5 cm wide and 3 cm long) that was not coated with activated carbon.

**Figure 1:**
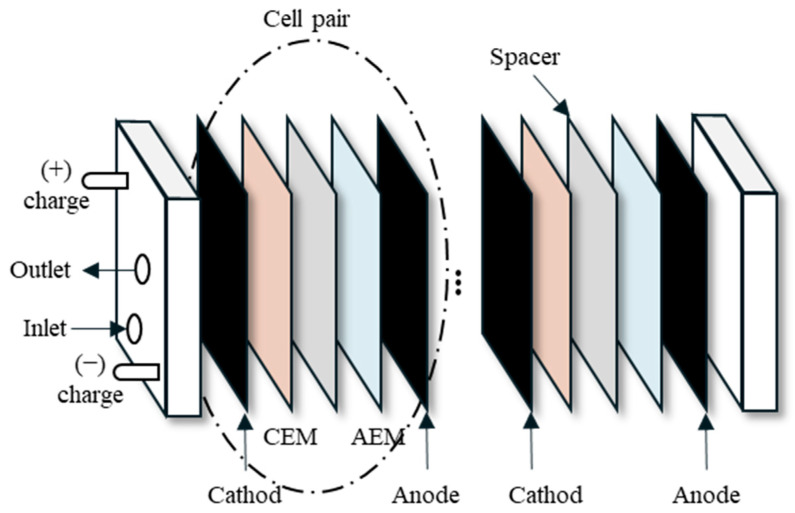
Schematic diagram of MCDI stack.

Figure 1 illustrates the configuration and sequence of the MCDI cell pairs, which are arranged in a specific order. Each cell pair consists of a cathode, a cation exchange membrane (CEM), a spacer, an anion exchange membrane (AEM), and an anode. A total of 20 cell pairs were assembled into a module. Holes 1 cm in diameter were drilled in the top left and center of the end plate of the MCDI module. These holes were made to allow for the inflow and outflow of water.

#### 2.2.2. MCDI Experimental Setup

**Figure 2:**
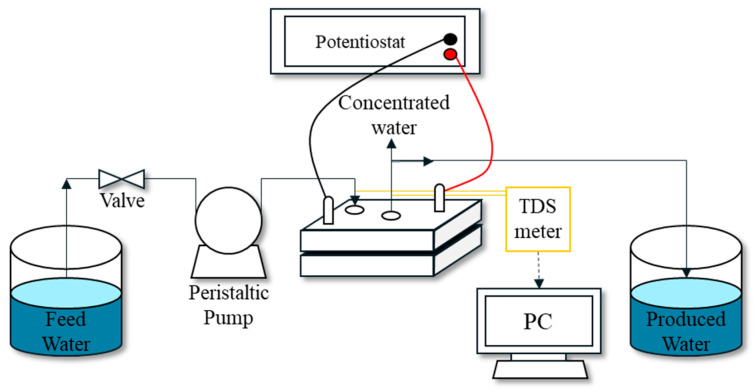
Schematic illustration of MCDI experimental setup.

Figure 2 shows a schematic of the MCDI setup for experiments. It includes the feed and product water tanks, a power supply, magnetic stirrers (C-MAG HS 7, IKA, Königswinter, Germany), and an MCDI cell. Furthermore, a peristaltic pump (7518-00 and 7551-00, Masterflex, Gelsenkirchen, Germany) is used to deliver a specified flow rate to the MCDI cell. A conductivity meter (CS150, Sensorex, Gardem Grove, CA, USA) was installed at the inlet and outlet of the MCDI cell to quantify the conductivity of the inflow and outflow.

### 2.3. Lab-Scale PRO System

#### 2.3.1. PRO Membranes

**Figure 3:**
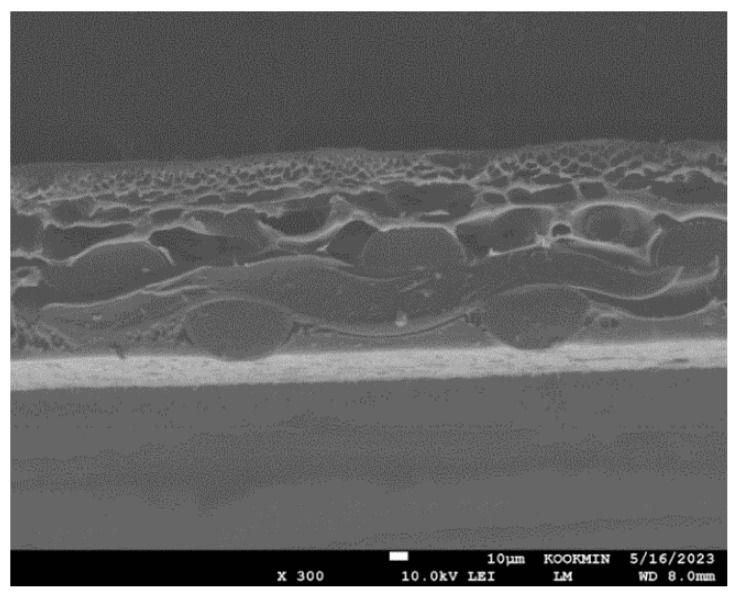
SEM images of the clean PRO membrane cross-section.

Thin-film composite (TFC) PRO membranes manufactured from Toray Chemical Korea were used in the experiments. As shown in Figure 3, the PRO membranes consist of a polyamide (PA) active layer and an embedded woven polymer support layer. According to the manufacturer, the water permeability and salt permeability of the PRO membranes are 1.97 L/m^2^-h-bar and 0.619 L/m^2^-h, respectively. Additional information on the PRO membranes is summarized in Table 3. Prior to testing, the membrane was stored in deionized water at a temperature below 4 °C.

#### 2.3.2. PRO Experimental Setup

**Figure 4:**
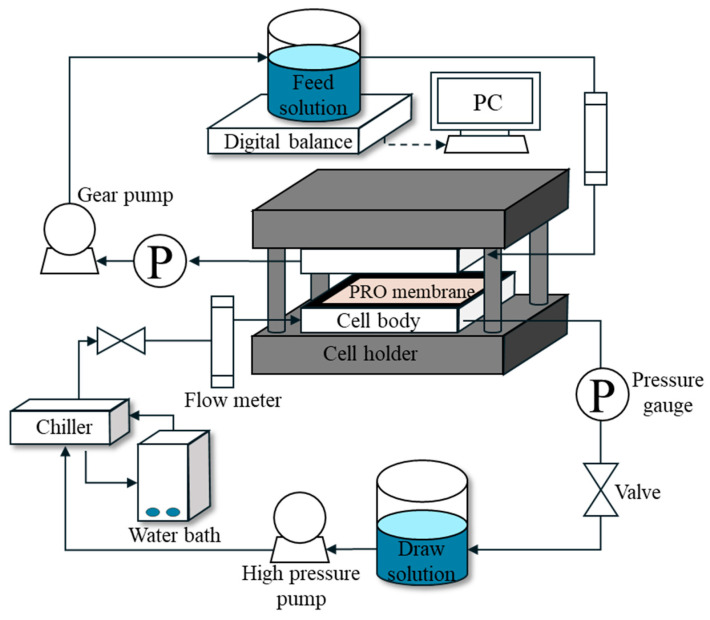
Schematic illustration of PRO experimental setup.

**Figure 5:**
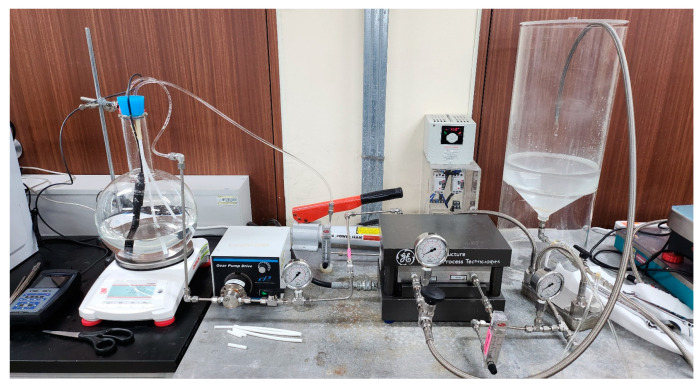
Photography of lab-scale PRO process.

Figure 4 shows a schematic of the bench-scale PRO system with the PRO Module. The process consists of a feed solution tank and a draw solution tank. A low-pressure pump (75211-15, Cole-Parmer, Chicago, IL, USA) was used to supply raw water to the feed solution side. On the draw solution side, a high-pressure pump (003XKSTHFECA, Hydra-cell pump, Upper Hutt, New Zealand) was connected to an inverter (IG5A, LS Electric, Anyang-si, Republic of Korea) to control the flow rate by adjusting the frequency. A plate-and-frame membrane cell holder (SEPA CF II, GE Infrastructure, Cincinnati, OH, USA) was used to examine the PRO membrane. The membrane cell, originally designed for RO membrane, was modified to operate in PRO mode. A mesh spacer was added on the feed side to prevent the PRO membrane from being damaged by the high pressure. An electronic balance (PX4202KR, OHAUS, Parsippany, NJ, USA) was installed on the feed solution side to measure the feed solution flow. The photography of the PRO system is presented in Figure 5.

The PRO membrane with an active area of 0.014 m^2^ (0.095 m × 0.145 m) was oriented in the AL (active layer)–DS (draw solution) mode, which is a typical membrane orientation for PRO processes [34]. The initial volumes of feed and draw solutions were 2 L and 4 L, respectively. The experimental conditions are summarized in Table 4.

### 2.4. Lab-Scale RED System

#### 2.4.1. Ion Exchange Membranes

Ion exchange membranes (IEMs) purchased from Fujifilm (Type-1, Fujifilm Manufacturing Europe, Tilburg, The Netherlands) were used in the experiments. The characteristics of these membranes are summarized in Table 5. The thicknesses of the CEM and AEM were 125 μm and 124 μm, respectively. Their surface resistances were 1.87 Ω-cm^2^ and 1.08 Ω-cm^2^, respectively. The transport coefficients for the CEM and AEM were 0.952 and 0.963, respectively. The IEMs are stacked between the electrodes. Similarly to MCDI cell pairs, RED cell pairs have a specific order consisting of a cathode, a CEM, a spacer, a seal, an AEM, and an anode. In these experiments, 10 and 20 cell pairs were stacked to form a module. Prior to the experiment, the ion exchange membranes were stored in 1 mM NaCl solution. Specifically, the CEMs were placed at the ends of the cell pairs to shield the membranes from the electrode wash solution (EDS).

#### 2.4.2. RED Experimental Setup

**Figure 6:**
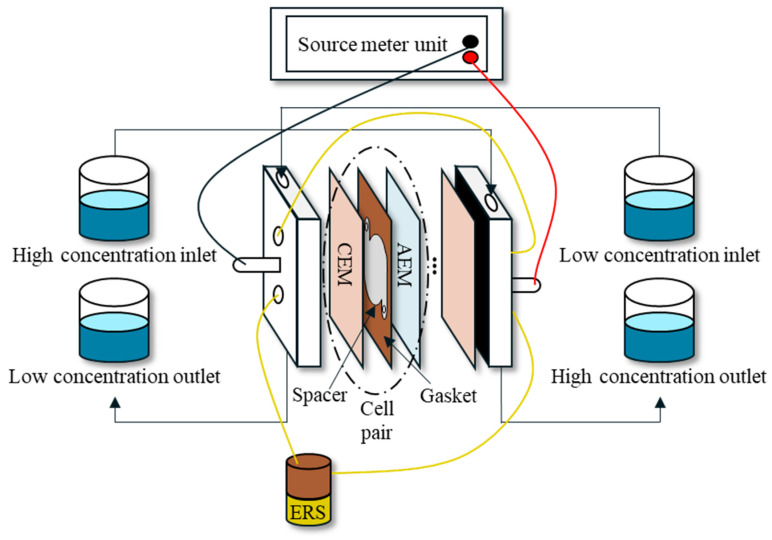
Schematic illustration of RED experimental setup.

**Figure 7:**
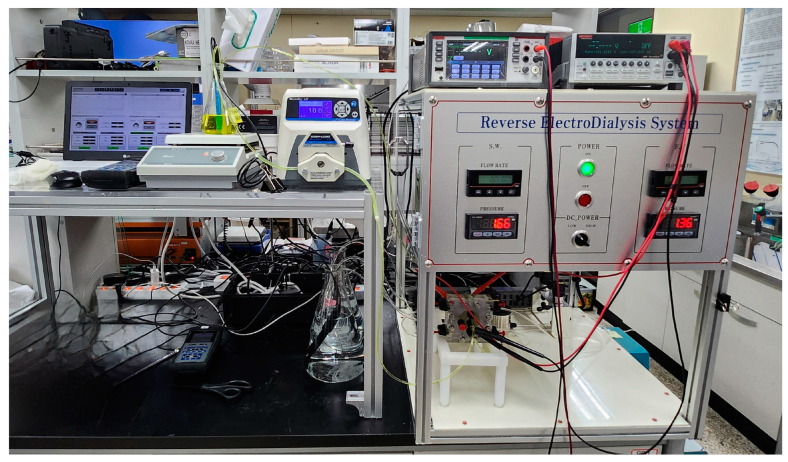
Photograph of the RED experimental setup.

The schematic of cell pairs and lab-scale RED experimental setup is presented in Figure 6. A photograph of the RED system is shown in Figure 7. The RED stack consists of two end plates made of acrylic resin which was provided by the Jeju Global Research Center, Korea Institute of Energy Research. Table 6 summarizes the specifications of the RED setup. The membrane area of the RED module was 0.0196 m^2^, and the intermembrane distance, defined by the thickness of the spacer and gasket, is 100 μm. The spacer and gasket were arranged in the RED module to prevent membrane contact and to provide a channel for the feed solution [35]. A smaller spacing between the membranes results in a higher gross power density [36]. The most common spacer thicknesses are 100, 200, and 300 μm, which are known to provide an optimal balance between power output and the pumping power input [37,38].

The electrode rinse solution (EDS), consisting of two redox species, is pumped and circulated from the anode to the cathode to facilitate energy generation in the RED process. In these experiments, [Fe(CN)_6_]^3−^/[Fe(CN)_6_]^4−^ at 50 mM was used as the redox pair, which is suitable for metallic electrodes [39]. Open circuit voltage (OCV) and power were measured with a source meter (Keithley 2401, Cleveland, OH, USA). Micro flow pumps (Masterflex, Gelsenkirchen, Germany) were used to maintain a low flow rate during the experiments.

### 2.5. Experimental Conditions

#### 2.5.1. MCDI Process

The operating conditions for the MCDI experiments are listed in Table 7. The feed solution used in these experiments is BWRO brine, as summarized in Table 1. To evaluate the effect of flow rate on MCDI process, the flow rates were varied in three steps: 10, 30, and 50 mL/min. They were referred to as MCDI 10, MCDI 30, and MCDI 50, respectively. The other parameters, including cell pairs, electrical potential, and cycle time, were fixed. These parameters were selected to reduce the removal efficiency of the MCDI process with BWRO brine. In these experiments, 20 cell pairs were stacked to secure the required contact area of the membranes. The electric potential was fixed at 1.5 V, which is the maximum voltage for the MCDI process. The cycle time was set to 300 s for both the charge and discharge phases, as commonly used before MCDI experiments. To reduce the load on the electrodes, a rest time of 10 s was included between these two phases. To obtain a volume of MCDI products suitable for the feed water of the PRO and RED process, each experiment was conducted for over 5 h. To maintain consistent water quality, the final conductivity and other water quality parameters of the MCDI products were measured after more than 5 h of operation.

#### 2.5.2. PRO Process

Prior to conducting the MCDI-PRO experiments, a set of preliminary experiments were conducted to investigate the effect of the hydraulic pressure on the basic performance of the PRO membrane. The hydraulic pressure applied on the draw solution side varied from 0 bar to 25 bar with 5 bar increments to perform the basic PRO process and determine the applied pressure. Details of these preliminary experimental conditions are summarized in Table 8. The feed and draw solutions were the deionized water and NaCl 1.2 M (70,200 mg/L), respectively, and their flow rates were 0.5 L/min and 1.0 L/min, respectively. The cross-flow velocities were calculated using the area of the feed spacer and the flow rate [40]. The water flux and power density of the PRO membrane were analyzed after one hour of stabilization time.

After the preliminary tests, the MCDI-PRO experiments were performed, and their conditions are summarized in Table 9. The hydraulic pressure for the PRO process was determined to be 15 bars based on the preliminary experimental results. Four different feed solutions were used in the MCDI-PRO process, including the BWRO brine and three types of MCDI products (MCDI 10, MCDI 30, and MCDI 50). Except for the conditions of the feed solutions and the pressure, the other parameters, including the membrane area, flow rate, and volumes of feed and draw solutions, were kept constant.

#### 2.5.3. RED Process

Prior to conducting the MCDI-RED experiments, the effect of the flow rates on the open circuit voltage (OCV) in the RED process was explored through a series of experiments. The corresponding conditions of these experiments are summarized in Table 10. The low TDS and high TDS solutions were DI water and 1.2 M NaCl solutions, respectively. The flow rates of the two solutions were adjusted from 10 to 100 mL/min in increments of 10 mL/min. The cross-flow velocities were calculated using the area of the feed spacer and the flow rate [40]. The other parameters were set to be constant.

The experimental conditions of the MCDI-RED process are summarized in Table 11. Similarly to the MCDI-PRO experimental cases, four different feed solutions were used as low TDS solutions in the MCDI-RED process: the BWRO brine and three types of MCDI products (MCDI 10, MCDI 30, and MCDI 50). The high TDS solution was a 1.2 M NaCl solution. The flow rates of both solutions were set at 10 mL/min based on the OCV test results. The cell pairs consisted of 10 stacks with the EDS flow rate fixed at 10 mL/min. The MCDI-RED experiments were run for 5 h to investigate power density degradation and membrane fouling during long-term operation.

### 2.6. Measurements and Observation

#### 2.6.1. Electrical Resistance of IEMs

The electrical resistance of IEMs, specifically membrane resistance, is a critical factor influencing the performance of the RED process because it directly impacted the maximum power output of RED [11,41]. Before the resistance measurement, the membrane was equilibrated by immersing it in a 0.5 g/L NaCl solution for at a minimum of 24 h. Typically, this resistance is assessed through impedance measurements using AC at a frequency of 1000 Hz. The membrane resistance (R_m_) was calculated by dividing the measured resistance (R_s_) by the membrane area (A) to obtain the resistance per unit area when the membrane and the solution are in contact. This conversion allows for the measured resistance value, based on the total membrane area, to be expressed as resistance per unit area. This relationship can be represented by the following equation [42]:

(1) $$R_{m}[\Omega \cdot {cm}^{2}]=\frac{R_{s}}{A}$$

#### 2.6.2. Power Density

Power density is a key indicator of the nature and dynamics of energy systems. In RED, power density was measured using a source meter (Keithley 2401, SnM Korea, Seongnam-si, Republic of Korea). In contrast, for PRO, power density (W) was calculated from the water flux ($J_{W}$) and hydraulic pressure (∆P) using the following equation [43,44]:

(2) $$J_{W}=A(∆\pi -∆P)$$

(3) $$W=J_{W}\times ∆P$$

where $J_{W}$ is the water flux, A is the water permeation coefficient of the membrane, ∆π is osmotic pressure difference, and ∆P is the hydraulic pressure difference in the PRO membrane. The power density for RED was calculated using the following equation [45]:

(4) W=VI/A

where V is the output voltage and I is the current.

#### 2.6.3. Specific Energy Consumption

Specific energy consumption (SEC) in MCDI is defined as the ratio of the applied electrical energy to the volume of the treated water. The amount of applied electrical energy was calculated by averaging current over charging time and multiplying by the voltage [46]. Since there are adsorption (charging) and desorption (discharging) cycles in MCDI, the energy consumption considering the energy recovery is expressed as presented below [31,47]:

(5) $$E_{in}=\int_{0}^{t}IVdt\;\;\;where\;IV>0$$

(6) $$E_{out}=\int_{0}^{t}IVdt\;\;\;where\;IV<0$$

(7) $$SEC=\frac{E_{in}-\eta E_{out}}{V}$$

where η is the energy recovery efficiency in the desorption step. Assuming perfect energy recovery, η becomes 1. In reality, η is between 0 and 1.

#### 2.6.4. Examination of Membranes Surface

Direct visual observation and scanning electron microscopy (SEM) were used to observe the surface of PRO and RED membranes. For direct visual observation, photographs were taken with a standard camera (Samsung, Suwon-si, Republic of Korea). In addition, a stereomicroscope (Leica S9D, Leica Microsystems, Wetzlar, Germany) equipped with a microscope camera (Flexacam C3, Leica Microsystems, Wetzlar, Germany) was used to examine the membrane surfaces. Field emission scanning electron microscopy (FE-SEM, SU8700, Hitachi, Tokyo, Japan) was used to analyze the membrane surfaces before and after the experiments. Prior to SEM analysis, the membrane samples were coated with a 5 nm layer of platinum for 40 s using an EMITECH SC 7620 sputter coater (Quorum Technologies Ltd., Lewes, UK). All samples were scanned at an accelerating voltage of 5 kV. SEM-EDS mapping images were obtained using the FE-SEM to identify contaminants and elements on the membrane surfaces.

## 3. Results and Discussions

### 3.1. Pretreatment of BWRO Brine by MCDI

MCDI was used as the pretreatment process with BWRO brine. Flow rates were set at 10 mL/min, 30 mL/min, and 50 mL/min. The water qualities of the treated water samples and the removal efficiencies are summarized in Table 12 and Table 13. The results show that ions and organics decreased as the flow rate increased. The highest ion removal, 88.8%, was achieved in MCDI 10. The ion removal in MCDI 30 was 81.4% and decreased to 69.5% in MCDI 50. Similarly, the TOC removal was 78.8% in MCDI 10. TOC removal decreased to 76.6% in MCDI 30 and further to 73.3% in MCDI 50. This decrease in removal is attributed to the reduced contact time between the electrode and the feed water due to the increased flow rate. Compared to the ion removals, the TOC removals were slightly lower. For example, the highest ion removal was 88.8%, while the highest TOC removal was 78.8%. Although the main purpose of the MCDI pretreatment was to partially remove ionic species, it could also remove organic matter due to electrostatic attraction [48], resulting in high-quality pretreated water.

### 3.2. PRO Experiments

#### 3.2.1. Flux and Power Density of PRO System

**Figure 8:**
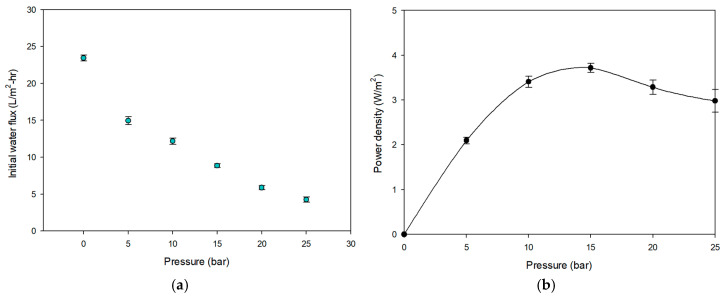
(**a**) Initial water flux; (**b**) initial power density on PRO process according to different pressures. The PRO test was performed using NaCl 1.2 M for HC and D.I. water as LC.

Using the feed waters in Table 8, the flux and power density in the PRO system were evaluated as a function of applied pressure under non-fouling conditions. Figure 8a shows the effect of pressure on flux. Since the driving force across the PRO membrane decreases as the pressure increases, the flux is reduced by the pressure. For example, the flux was 24.4 L/m^2^-h at 0 bar and decreased to 4.7 L/m^2^-h at 25 bars, a reduction of 80.7%. The power density calculated using Equation (3) is shown in Figure 8b. The maximum power density (3.79 W/m^2^) is at 15 bars of pressure. In an ideal PRO system, the power density reaches its maximum at 50% of the osmotic pressure difference between the feed and draw solutions, which should be around 27 bars. This deviation can be attributed to the compaction of the PRO membrane above the 15-bar pressure. Compaction not only reduces the thickness of the support layer but also changes the porosity and tortuosity as the pores collapse [48], leading to a reduction in flux and power density. The manufacturer also indicated that the membrane may not be stable under high applied pressure. Accordingly, the applied pressure for the MCDI-PRO experiment was set at 15 bars.

#### 3.2.2. Fouling in PRO System

**Figure 9:**
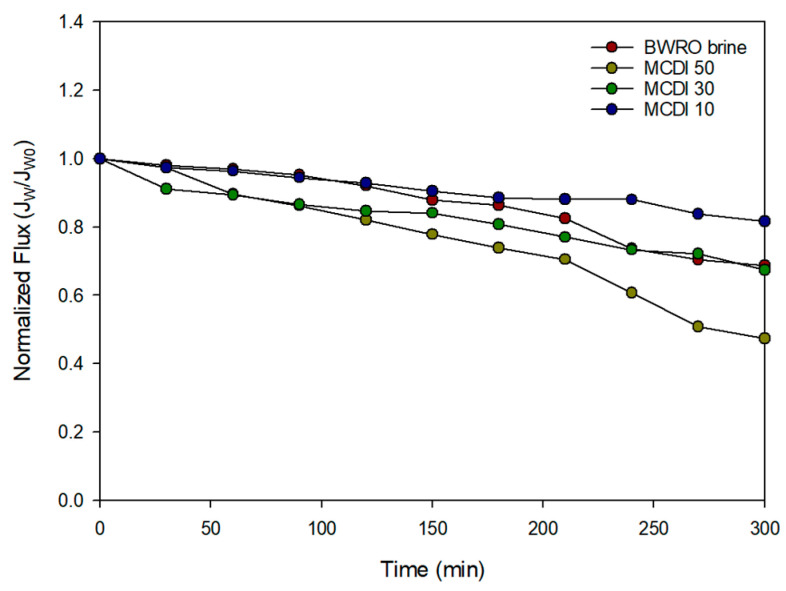
Normalized PRO water flux for MCDI 10, MCDI 30, MCDI 50, and BWRO brine.

A set of experiments were conducted in the PRO system using the feed and draw solutions in Table 9. Figure 9 illustrates the normalized flux (*J*/*J*_0_) over time for different feed solutions, including BWRO brine and three MCDI-treated brines: MCDI 10, MCDI 30, and MCDI 50. This normalization method is consistent with approaches used in the existing literature on membrane fouling [43,49,50,51]. The initial flux was normalized to 1.0 for all cases to enable comparison. The flux decline observed across all samples indicates the degree of fouling or scaling during membrane operation. Among the tested solutions, the BWRO brine exhibits the most pronounced flux decline, suggesting a higher fouling tendency. In contrast, MCDI-treated brines demonstrate improved performance, with less flux reduction over the experimental duration. Notably, the sample treated with MCDI 10 maintained the highest flux retention, indicating superior mitigation of fouling or scaling. These results highlight the effectiveness of MCDI treatment in reducing brine fouling potential, with lower flow rates leading to better performance.

### 3.3. RED Experiments

#### 3.3.1. OCV of RED System

Table 14 provides the OCV as a function of flow rate and NaCl concentration in the high TDS solution in the RED system. Details of the experimental conditions are given in Table 10. The results showed that the OCV ranged from 1.78 V to 1.97 V. When the flow rate was 10 mL/min, the OCV was the lowest. At a flow rate above 20 mL/min, the OCV was above 1.90 V. Based on these results, the flow rate for the MCDI-RED experiment was set at 10 mL/min because the OCV values above 20 mL/min were similar.

#### 3.3.2. Power Density of RED System

**Figure 10:**
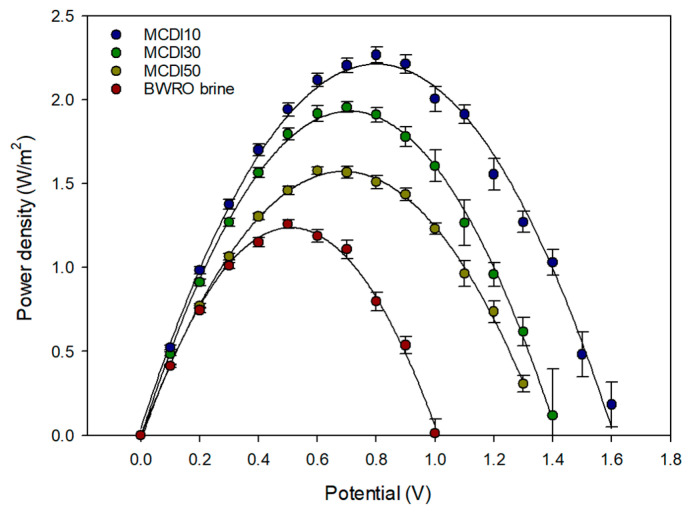
Voltage power density curve of BWRO brine and MCDI-RED process.

Figure 10 depicts the power density in the RED system as a function of applied potential for the BWRO brine and the MCDI-treated brines: MCDI 10, MCDI 30, and MCDI 50. The power density shows a parabolic trend for all samples, peaking at an intermediate potential before declining to higher potentials due to increased resistive effects. Among the samples tested, MCDI 10 exhibits the highest peak power density, followed by MCDI 30 and MCDI 50, while the BWRO brine exhibits the lowest power density over the entire potential range. These results indicate that fewer MCDI cycles improve the electrochemical performance of the treated brine, likely due to enhanced ion removal and reduced fouling, thereby improving conductivity and reducing internal resistance. Error bars representing the standard deviation highlight the reproducibility of the measurements. The comparison underscores the significant improvement in power generation achievable with MCDI treatment, especially with low flow rates. Since the voltage at maximum power was approximately half of the OCV, the applied voltages were determined based on the experimental results: BWRO brine, MCDI 10, MCDI 30, and MCDI 50, in that order, were 0.6 V, 0.8 V, 0.8 V, and 0.7 V, respectively.

#### 3.3.3. Electrical Resistance of RED System

**Figure 11:**
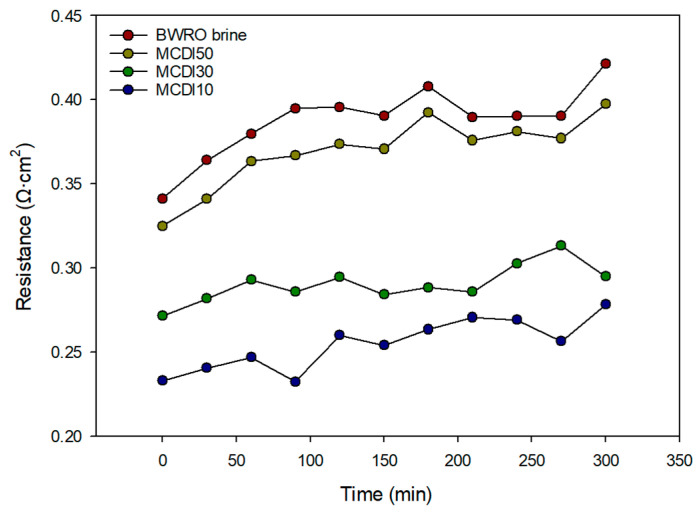
Variation in the electrical resistance at the four different feed solutions in RED process (BWRO brine, MCDI50, MCDI30, MCDI10).

Figure 11 shows the change in resistance over time for BWRO brine and three MCDI-treated brines. The resistance of the BWRO brine steadily increases over time, reaching the highest values, compared to the MCDI-treated brines, indicating significant fouling effects. Among the MCDI-treated samples, MCDI 10 consistently exhibits the lowest resistance throughout the duration of the experiment, followed by MCDI 30 and MCDI 50. This trend suggests that the MCDI process with low flow rates is more effective at maintaining lower resistance by reducing fouling in the brine solution. The observed resistance behavior directly correlates with the improved flux and power density performance of the MCDI-treated brines shown in previous figures. These results highlight enhanced electrochemical performance and operational stability achievable by optimizing the MCDI cycle parameters.

### 3.4. Comparison of Power Density Between PRO and RED

#### 3.4.1. PRO System

**Figure 12:**
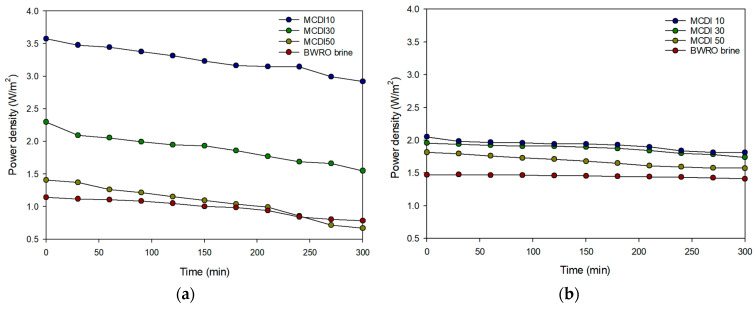
Results of power density during long-term operation: (**a**) MCDI-PRO; (**b**) MCDI-RED.

The effect of the MCDI pretreatment on the power density of the PRO system was investigated, and the results are shown in Figure 12a. The initial power density for MCDI 10 was the highest, reaching 3.57 W/m^2^, which is three times the initial power density for the BWRO brine (1.14 W/m^2^). This improvement is attributed to an increased salinity gradient between the draw solution and the feed solution in MCDI 10. As shown in Table 12, the electrical conductivity removal for MCDI 10 was 88.8%, resulting in an increase in the salinity difference across the PRO membrane. The initial power densities for MCDI 30 and MCDI 50 were also higher than that of the BWRO brine but lower than that of MCDI 10.

The reduction ratio, calculated from the initial and final values after 5 h, improved from 31.3% to 18.3%. However, the lowest power density was observed for MCDI 50 with 1.41 W/m^2^. This result was closest to the initial power density of the BWRO brine, and the final power density decreased relative to that of the BWRO brine. The rate of power density reduction was calculated to be the highest among the feed solution conditions at 52.6%, and this decrease is attributed to membrane fouling caused by the accumulation of organic and inorganic matter in the feed solution. Despite the pretreatment in the MCDI process, the rapid flow rate resulted in a decrease in the removal rate and did not mitigate the decrease in power density caused by membrane fouling.

#### 3.4.2. RED System

Figure 12b shows the changes in power density in the RED system under different pretreatment conditions. Over a period of 5 h of operation, the power density increased from 1.47 W/m^2^ with BWRO brine to 2.05 W/m^2^ with MCDI 10, which is the lowest concentration of the three MCDI products. Similarly to the PRO system, this increase is due to an increased salinity gradient. Although other conditions also resulted in improvements in power density, these were less pronounced than those observed with MCDI 10. In addition, the rate of power density reduction worsened from 4.1% in BWRO brine to 11.7% in MCDI 10. Among the three types of MCDI products evaluated, MCDI 50, which has the highest concentration, showed the greatest reduction in power density at 13.3%.

#### 3.4.3. Comparison of MCDI Pretreatment Efficiency for PRO and RED

When comparing the power density improvements over BWRO brine, MCDI-RED process demonstrated enhancement by 1.4 times with MCDI 10, while the MCDI-PRO process achieved improvement by three times. These results are consistent with existing research indicating that the power density of PRO generally surpasses that of RED [20]. However, under the MCDI 50 condition, the reduction rate in power density for MCDI-PRO was markedly higher at 52.6%, compared to 13.3% for MCDI-RED. This significant reduction for MCDI-PRO suggests that the thin-film composite membrane is more vulnerable than ion exchange membranes to fouling. This can also be attributed to the difference in water flux between PRO and RED. As shown in Figure 9, the water flux in PRO ranges from 4.5 L/m^2^-h to 9 L/m^2^-h. On the other hand, the water flux in RED is negligible because only ions move through the ion exchange membranes. Since higher flux generally results in more rapid fouling, PRO appears to have a higher fouling tendency than RED.

### 3.5. Examination of PRO and RED Membranes

#### 3.5.1. Visual Examination of PRO Membranes

**Figure 13:**
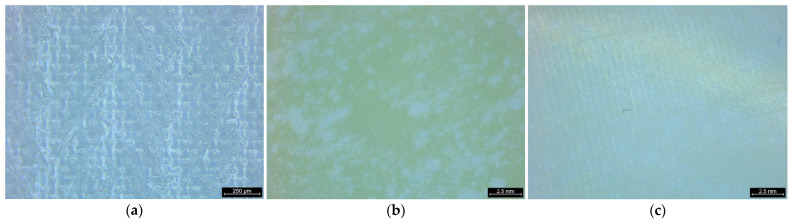
Visual observation of PRO membranes with a stereo microscope: (**a**) pristine membrane; (**b**) after BWRO brine; (**c**) after MCDI product.

Figure 13 presents stereo microscope images of PRO membranes under different conditions: (a) pristine membrane, (b) membrane after the use of BWRO brine, and (c) membrane after the use of MCDI-treated product. The pristine membrane (Figure 13a) exhibits a clean and uniform surface texture with no visible fouling. In contrast, the membrane after the use of the BWRO brine (Figure 13b) shows significant foulant deposits, resulting in a visibly rough and uneven surface. This observation highlights the detrimental effect of untreated brine on membrane performance due to fouling. Since the yellow foulants from the BWRO brine are mainly organic, the results indicate that the fouling is associated with organic compounds present in the BWRO brine. On the other hand, the membrane after the use of the MCDI product (Figure 13c) displays a relatively smooth surface with minimal fouling compared to the case with BWRO brine. These visual observations confirm the effectiveness of the MCDI pretreatment in mitigating fouling on PRO membranes, enhancing their operational stability and performance.

#### 3.5.2. SEM Analysis of PRO Membranes

**Figure 14:**
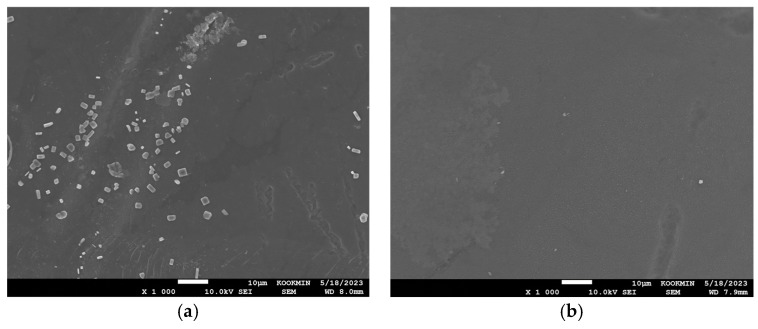
SEM image on PRO membranes: (**a**) BWRO brine; (**b**) after MCDI product.

SEM images of PRO membranes are shown in Figure 14. When the BWRO brine was used in the PRO system (Figure 14a), noticeable deposits are observed within the support layer, indicating significant foulant accumulation. The fouling appears to obstruct the membrane’s porous structure, potentially reducing water flux and power density. Conversely, when the MCDI product was used (Figure 14b), a much cleaner and more intact structure was shown with reduced deposits in the support layer.

#### 3.5.3. Visual Examination of RED Membranes

**Figure 15:**
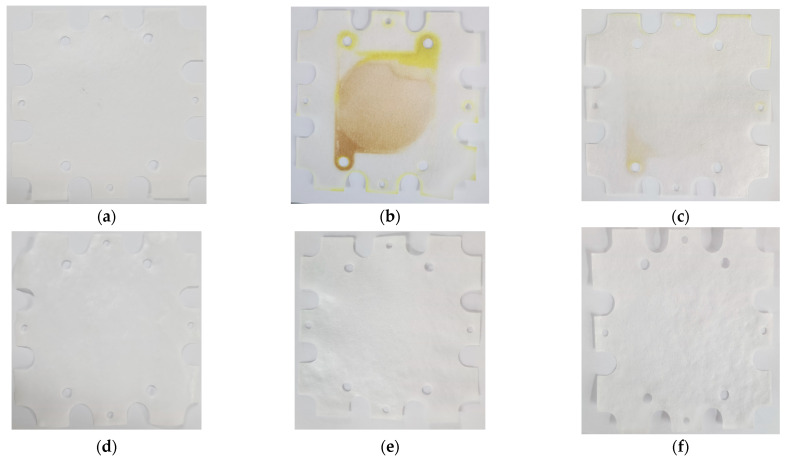
Visual observation of IEMs: (**a**) pristine AEM; (**b**) after BWRO brine AEM; (**c**) after MCDI product AEM; (**d**) pristine CEM; (**e**) after BWRO brine CEM; (**f**) after MCDI product CEM.

The ion exchange membranes (IEMs) were visually examined after the RED experiments with the BWRO brine and the MCDI-treated products. The pristine anion exchange membrane (AEM) and cation exchange membrane (CEM) are shown in Figure 15a and 15d, respectively, both displaying a clean, uniform surface. In contrast, the AEM with the BWRO brine (Figure 15b) exhibits significant discoloration and fouling, indicating a severe deposition of foulants and organic/inorganic impurities. The AEM with the MCDI product (Figure 15c) appears relatively clean with only minimal fouling. Compared with the AEMs, the deposition of foulants on the CEMs was negligible. Both the CEMs with the BWRO brine (Figure 15e) and with the MCDI product (Figure 15f) retain a clean surface with negligible deposits. These results suggest that fouling occurred exclusively in AEM due to the characteristics of organic substances. Generally, the movement of organic matter toward the AEM was influenced by the negatively charged nature of organics including both hydrophobic and hydrophilic materials in the feed, when an electric field was applied [52].

#### 3.5.4. SEM Analysis of RED Membranes

**Figure 16:**
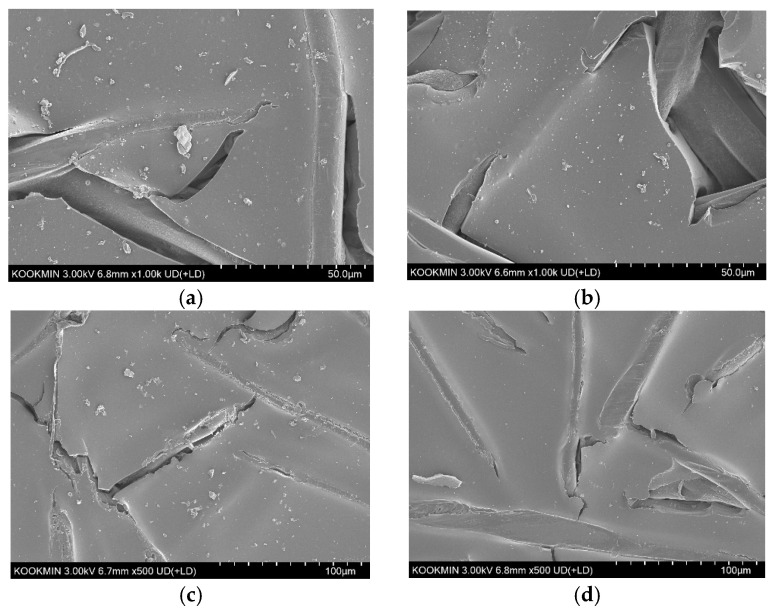
SEM image of IEMs: (**a**) AEM with BWRO brine; (**b**) AEM with MCDI product; (**c**) CEM with BWRO brine; (**d**) CEM with MCDI product.

The SEM images of the ion exchange membranes (IEMs) after the RED experiments are shown in Figure 16. The AEM with the BWRO brine (Figure 16a) shows the presence of deposits indicative of fouling. In contrast, the AEM with the MCDI product (Figure 16b) shows less visible deposits, indicating effective fouling control by the MCDI pretreatment. On the other hand, the foulant deposits on the CEMs were less significant than those on the AEMs (Figure 16c,d). After the MCDI pretreatment, the amounts of deposits on the CEM became almost negligible.

### 3.6. Energy Efficiency

#### 3.6.1. Specific Energy Consumption for MCDI

**Figure 17:**
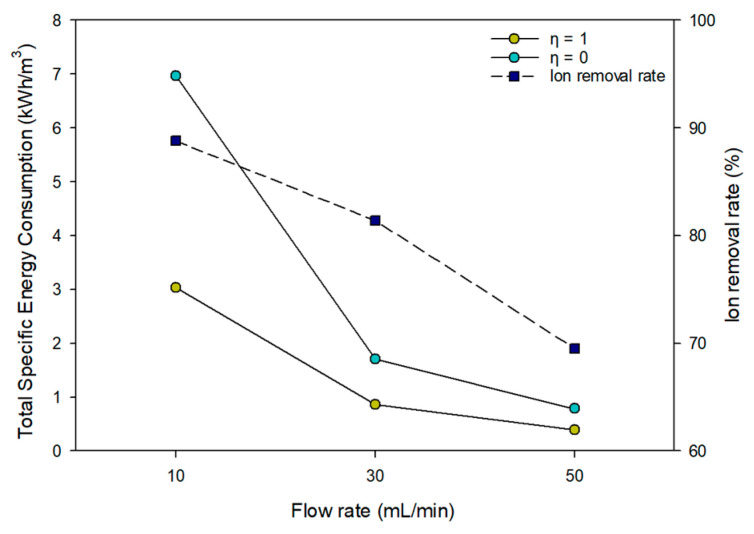
Effect of flow rates on ion removal rate and total SEC (e.g., η=0, η=1).

The ion removal rate and energy consumption (SEC) of MCDI at various flow rates (10–50 mL/min) are illustrated in Figure 17. The ion removal rate of MCDI decreased as the flow rates increased, reaching a maximum of 88.8% at 10 mL/min. Since the volume of produced water was fixed at 2 L, the total operating time varies based on the flow conditions, and the production capacity adjusts accordingly. Therefore, 20 cycles were conducted for MCDI10, 7 cycles for MCDI30, and 4 cycles for MCDI50. SEC was derived by calculating V_d_. The results indicated SEC of 3.03 kWh/m^3^ for MCDI10, which had the longest duration, 0.86 kWh/m^3^ for MCDI30, and 0.39 kWh/m^3^ for MCDI50, assuming energy recovery (e.g., η=1). These results align with existing literature, which indicates that energy consumption for typical MCDI processes ranges from 0.5 to 3 kWh/m^3^. Furthermore, the findings demonstrate that the ion rejection rate is higher under lower flow conditions, while energy efficiency improves at higher flow rates. In the case of no energy recovery (e.g., η=0), the total SEC calculated ranged from 6.97 kW/h/m^3^ to 0.78 kW/m^3^. The results indicated that, despite being more removal efficient, the MCDI was less energy efficient when operated at lower flow rates.

For comparison, Table 15 shows the SEC results of this study with the pilot-scale CDI SEC results. The SEC results from this study, which assumed the use of an energy recovery device, indicate lower energy consumption compared to the SEC results from previous studies. This finding suggests that process optimization can lead to more efficient operating performance.

#### 3.6.2. Specific Energy Generation

The net energy generated by MCDI-PRO or MCDI-RED is calculated using the following equation:

(8) $${SEG}_{net}={SEG}_{theory}\varepsilon -{SEC}_{MCDI}$$

where SEG is the net specific energy, SEG_theory_ is the theoretical maximum specific energy generation, ε is the ratio of actual to theoretical energy generation, and SEC_MCDI_ is the specific energy consumption of the MCDI pretreatment. Assuming that the SWRO brine is used, the SEG_theory_ is the theoretical salinity gradient power, which is approximately 53.5 bars or 1.446 kWh/m^3^. To make SEG_net_ positive, SEC_MCDI_ should be less than SEG_theory_, which is possible for MCDI 50. Since ε is always less than 1, it is necessary to reduce SEC_MCDI_ in the future. A possible solution may be the integration of MCDI with renewable energy sources [55,56], but further work should be carried out for an in-depth investigation of these issues.

## 4. Conclusions

This study demonstrates the effectiveness of MCDI as a pretreatment strategy to SGP generation through PRO and RED. The experimental results show that MCDI significantly improves the power density of both systems by mitigating fouling and scaling caused by organic and inorganic substances present in brackish water reverse osmosis (BWRO) brine. In particular, MCDI pretreatment with a low flow rate (MCDI 10) yielded the highest power density improvements, reaching 3.57 W/m^2^ in PRO and 2.05 W/m^2^ in RED, representing a 3-fold and 1.4-fold increase, respectively, compared to untreated BWRO brine. Despite the enhanced power generation and operational stability, further optimization is required to minimize the energy consumption of MCDI while maintaining high removal efficiency. The integration of MCDI with renewable energy sources or energy recovery systems could improve the overall feasibility and sustainability of the hybrid MCDI-PRO and MCDI-RED processes. Future studies should focus on conducting techno-economic analyses to determine the scalability and long-term viability of this approach in real-world applications.

## Figures and Tables

**Table 1:** Summary of water quality in BWRO brine from wastewater reuse plant.

Parameters		Unit	Conditions
TOC		mg/L	49.6
UV-254		cm^−1^	0.30
SS		mg/L	17.7
pH		-	8
Electric conductivity		μS/cm	5250
Cations	Na^+^	mg/L	706.5
	K^+^		108.3
	Ca^2+^		339.6
	Mg^2+^		37.6
Anions	F^−^		6.8
	Cl^−^		941.2
	SO_4_^2−^		1269.3
	NO_3_^−^		58.2

**Table 2:** Characteristics of MCDI module and ion exchange membrane.

		Material	Standard	Specification
Electrode		Activated Carbon, Graphite	W 100 × L 100 × T 0.6 mm^3^	Capacity: >16 mg/g (2000 mg/L as NaCl, 0.1 mL/cm^2^∙min) Density: 0.6 ± 0.1 g/cm^3^
Ion exchange membrane ^1^	CEM	Polyethylene	W 100 × L 100 × T 0.015 mm^3^	Capacity: >1.6 meq/g (Sheet resistance: <0.5 Ω cm^2^)
	AEM			Capacity: >1.7 meq/g (Sheet resistance: <0.3 Ω cm^2^)
Spacer		Polyethylene terephthalate	W 110 × L 110 × T 0.1 mm^3^	Mesh opening: 0.2 mm Tread diameter: 0.055 mm Mesh count: 99 mesh/inch

^1^ Specific details regarding the ion exchange membranes remain confidential to the manufacturer.

**Table 3:** Properties of PRO membrane.

Conditions	Unit	Values
Manufacture	-	CMS-PRO-4 (Toray, Seoul, Republic of Korea)
Water permeability, A	L/m^2^·h·bar	1.97
Salt (NaCl) permeability, B	L/m^2^·h	0.619
Structural parameter, S	μm	713

**Table 4:** Experimental setup of lab-scale PRO process.

Conditions		Values
Membrane type		Thin-film composite (TFC) PRO membrane
Effective membrane area		$0.014\;m^{2}\;(0.095\;m\;\times$ 0.145 m)
Membrane orientation		AL-DS (PRO mode)
Flow rate	Feed solution	0.5 L/min
	Draw solution	1.0 L/min
Initial volume	Feed solution	2 L
	Draw solution	4 L

**Table 5:** Properties of ion exchange membranes, spacer, and gasket of RED.

		Manufacture	Material	Thickness	Area Resistance	Perm Selectivity
IEMs	CEM	Fujifilm (Type-1), Tilburg, The Netherlands	polyolefin	135 μm	2.7 Ω·cm^2^	92
	AEM			125 μm	1.3 Ω·cm^2^	
Spacer		DS mesh, Seoul, Republic of Korea		100 μm (mesh with 81.3% open area)		
Gasket		Tommy hecco, Seoul, Republic of Korea	PTFE	100 μm		

**Table 6:** Experimental setup of lab-scale RED process.

Specifications	Values
Area of one membrane	0.0196 m^2^ (14 cm × 14 cm)
Membrane pairs	20 pairs
Intermembrane distance	100 μm
Electrode rinse solution	[Fe(CN)_6_]^3−^/[Fe(CN)_6_]^4−^ 50 mM
Initial volume	1 L (both HC, LC)

**Table 7:** Operating conditions of MCDI process.

Parameters		Unit	Values		
Notation			MCDI 10	MCDI 30	MCDI 50
Flow rate		mL/min	10	30	50
Feed solution			BWRO brine (refer to Table 1)		
Cell pairs		Stack	20		
Area of one membrane		cm^2^	100		
Electric potential		V	±1.5		
Cycle	Charge/Discharge	s	300		
	Rest		10		
Operation time		h	over 5		

**Table 8:** Conditions of applied pressure for optimization in the PRO system.

Parameters		Values					
Pressure (bar)		0	5	10	15	20	25
Feed Solution	Type	Deionized water					
	Flow rate Cross-flow velocity	0.5 L/min 23 cm/s					
	Volume	2 L					
Draw Solution	Type	NaCl 1.2 M (70,200 mg/L)					
	Flow rate Cross-flow velocity	1.0 L/min 11.5 cm/s					
	Volume	4 L					

**Table 9:** Conditions of MCDI-PRO system.

Parameters		Values			
Pressure (bar)		15			
Feed Solution	Type	BWRO brine	MCDI 10	MCDI 30	MCDI 50
	Flow rate Cross-flow velocity	0.5 L/min 23 cm/s			
	Volume	2 L			
Draw Solution	Type	NaCl 1.2 M (70,200 mg/L)			
	Flow rate Cross-flow velocity	1.0 L/min 11.5 cm/s			
	Volume	4 L			

**Table 10:** Various parameters affecting the open circuit voltage (OCV) of RED system.

Parameters		Values									
Low TDS Solution	Type	DI water									
	Volume	1 L									
High TDS Solution	Type	NaCl 1.2 M (70,200 mg/L)									
	Volume	1 L									
Flow rate (mL/min) Cross-flow velocity (cm/s)		10 0.12	20 0.24	30 0.36	40 0.48	50 0.60	60 0.71	70 0.83	80 0.95	90 1.07	100 1.19
EDS flow rate		10 mL/min									
Cell pairs		10 stacks									

**Table 11:** Analysis of power density behaviors in MCDI-RED process.

Parameters		Values			
Low TDS Solution	Type	BWRO brine	MCDI 10	MCDI 30	MCDI 50
	Volume	1 L			
High TDS Solution	Type	NaCl 1.2 M (70,200 mg/L)			
	Volume	1 L			
Flow rate Cross-flow velocity		10 mL/min 0.12 cm/s			
EDS flow rate		10 mL/min			
Cell pairs		10 stacks			
Operation time		5 h			

**Table 12:** Summary of water quality and removal rates in BWRO brine and MCDI products.

Parameters	Unit	BWRO Brine	MCDI 10		MCDI 30		MCDI 50	
		a ^1^	a	b ^1^	a	b	a	b
				%		%		%
TOC	mg/L	49.6	10.5	78.8	11.6	76.6	13.3	73.2
UV-254	cm^−1^	0.30	0.07	76.7	0.07	76.7	0.08	73.3
SS	mg/L	17.7	3.7	79.1	4	77.4	5	71.8
Electric conductivity	μS/cm	5250	589	88.8	979	81.4	1602	69.5

^1^ a represents concentration, while b denotes the removal rate for each condition.

**Table 13:** Summary of water quality in BWRO brine and MCDI products.

Parameters		Unit	BWRO Brine	MCDI 10		MCDI 30		MCDI 50	
			a ^1^	a	b ^1^	a	b	a	b
					%		%		%
Cations	Na^+^	mg/L	706.5	123.6	82.5	155.3	78.0	165.0	76.6
	K^+^		108.3	15.6	85.6	23.1	78.7	23.9	77.9
	Ca^2+^		339.6	35.2	89.6	68.6	79.8	74.4	78.1
	Mg^2+^		37.6	4.3	88.6	8.4	77.7	8.9	76.3
Anions ^2^	F^−^		6.8	-	-	-	-	-	-
	Cl^−^		941.2	99.5	89.4	124.8	86.7	263.3	72.0
	SO_4_^2−^		1269.3	194.3	84.7	225.8	82.2	331.1	73.9
	NO_3_^−^		58.2	8.3	85.7	-	-	16.4	71.8

^1^ a represents concentration, while b denotes the removal rate for each condition. ^2^ F^−^ was not detected, and NO_3_^−^ was neither detected nor analyzed at low concentrations.

**Table 14:** Comparing with effect of different parameters on OCV in RED system.

	C_HC_	NaCl 1.2 M
Flow Rate		
10 mL/min		1.78
20 mL/min		1.91
30 mL/min		1.90
40 mL/min		1.95
50 mL/min		1.92
60 mL/min		1.97
70 mL/min		1.93
80 mL/min		1.97
90 mL/min		1.93
100 mL/min		1.95

**Table 15:** Comparison of configurations of MCDI configurations.

Type	C_i_ (g/L)	Treatment Capacity(m^3^/d)	Cell Voltage (V)	Energy Consumption (kWh/m^3^)	Ref.
MCDI	3.0	0.036	1.5	0.39 (0.78 no energy recovery)	(This study)
CDI	2.1	0.002	-	0.60	[53]
FCDI	2.0	0.0012	-	0.50–0.56	[54]
MCDI	1.10–4.65	0.043	1.4	0.17–3.45	[47]

## Data Availability

The original contributions presented in this study are included in the article. Further inquiries can be directed to the corresponding author.
